# Androgens Involvement in the Pathogenesis of Renal Stones Formation

**DOI:** 10.1371/journal.pone.0093790

**Published:** 2014-04-02

**Authors:** Mohammad Reza Naghii, Mnasour Babaei, Mehdi Hedayati

**Affiliations:** 1 Sport Physiology Research Center and Department of Nutrition, Health School, Baqiyatallah (a.s.) University of Medical Sciences, Tehran, Islamic Republic of Iran; 2 Medical Sciences Research Management, Health Management Research Center, Baqiyatallah (a.s.) University of Medical Sciences, Tehran, Islamic Republic of Iran; 3 Cellular and Molecular Endocrine Research Center, Research Institute for Endocrine Sciences, Shahid Beheshti University of Medical Sciences, Tehran, Islamic Republic of Iran; Baylor College of Medicine, United States of America

## Abstract

**Objective:**

The potential role for the gonadal steroids in the pathogenesis of urolithiasis, higher mean of plasma oxalate concentration and kidney calcium oxalate deposition influenced by androgens in men has been proposed. In this study, the serum levels of steroid hormones as a pathogenesis of this condition in male patients with active renal stone disease compared with controls was investigated.

**Methods:**

Forty patients diagnosed with renal stones and hospitalized for further clinical treatments or referred to our office after ultrasonographic evaluations participated in the study. Forty six healthy subjects served as controls. Steroid sex hormones in the plasma samples including testosterone, free testosterone, dihydrotestosterone, estradiol, and sex hormone binding globulin were analyzed.

**Results:**

A significant difference was observed between patients and the control subjects regarding serum testosterone, free testosterone, dihydrotestosterone, estradiol, and sex hormone binding globulin.

**Conclusions:**

Based on the results, a higher androgens level was diagnosed in renal stone patients, indicating a possibility of a substantial pathogenic role of testosterone, free testosterone, and dihydrotestosterone involvement in the pathogenesis of renal stones formation. Therefore, data presentation and further investigation on the relation between male steroids and urolithiasis is of importance and should be considered in evaluation of the etiology of the disease.

## Introduction

Urolithiasis is a multifactorial disorder and a complex process that is a consequence of an imbalance between promoters and inhibitors in the kidney [1]. Males have a three times higher incidence compared to females, indicating that sex hormones influence urinary stone formation [2]. Urolithiasis mainly occurs in the third and fourth decades of life when the level of serum testosterone is also the highest [3].

As one of the early factors in urinary stone pathogenesis, hormones can modulate their effect through changes in their serum levels, or in the sensitivity or activity of their receptors [4]. Recently, the potential role for the gonadal steroids in the pathogenesis of urolithiasis in male sex was proposed [5], the relationship of kidney calculi with high plasma total and free testosterone was reported [6] and castration in males was also associated with lower urinary oxalate excretion [7]. Kato et al. (2005) concluded that menopausal women might have an increased potential for urinary stone formation compared with premenopausal women. This female condition of low estrogen resembles the male hormonal status [8].

Data suggest that testosterone appears to promote stone formation by suppressing osteopontin expression in the kidneys and increasing urinary oxalate excretion, while estrogen appears to act inversely [9]. It is postulated that lower serum testosterone levels is regarded as protective for women and children against oxalate stone disease [1]. In contrast, it is reported that higher mean of plasma oxalate concentration and kidney calcium oxalate deposition in men are influenced by androgens [10], [11].

Although, the result of a study reported that serum levels of estradiol and testosterone were not statistically different between the male active renal calcium stone formers and control groups, the possibility of testosterone involvement in the pathogenesis of renal stones through higher urinary uric acid and oxalate excretion was postulated [4]. Testosterone is known to increase the hepatic levels of glycolic acid oxidase (GAO), an important enzyme in the metabolic pathway for urinary oxalate synthesis resulting in hyperoxaluria [12]. Urinary oxalate excretion increased 12.8-fold after 4 weeks of EG treatment, and it was concluded that dihydrotestosterone (DHT) was partially responsible for the observed exaggerated hyperoxaluria [13].

In a recent study, after EG exposure to induce urolithiasis in male rats, a positive trend was observed between high plasma androgen concentrations and incidence of kidney stones, indicating a potential role for the gonadal steroids in the pathogenesis of urolithiasis [14].

To elucidate the role of high steroid levels as a risk factor in kidney stone formation, further investigation on the relation between male steroids and urolithiasis is of importance and should be considered in evaluation of the etiology of the disease. Since, clinical proof for this hypothesis is limited; the pathogenesis of this male predisposition still remains to be elucidated and requires a larger prospective study.

Thus, the aim of this study was to investigate the serum levels of steroid hormones in male patients with active renal stone disease compared with controls.

## Materials and Methods

Adult males 21–60 years agreed to participate and were enrolled. They were diagnosed with renal lithiasis/urolithiasis and hospitalized in Baqiyattallah-alazam Hospital, Baqiyattallah University of Medical Sciences for further clinical treatments, or referred to our office after sonographic evaluations and confirmations of the stone formation. Of the participants, 40 patients and 46 healthy control subjects participated in the study. The controls were selected from similar age range with no positive history or episode of kidney or urinary stone complications during the previous 10 years. They were included in the control group after sonography evaluations revealed no complications in their renal system.

The study was approved by the Baqiyattallah University of Medical Sciences' Research and Ethics Committee. All participants signed the provided written informed consent to participate in this study. The above ethics committees approved the consent form.

The sonographic evaluations of the kidneys and urinary tract systems and the diagnosis and detection of renal lithiasis/urolithiasis were performed by the attending expert radiologists in the Department of Radiology and Sonography who were blinded to the clinical evaluations or the aim of this study at the time of examinations.

For hormone analysis, all samples of blood were collected at 8.00 A.M, and each sample was centrifuged at 3000 g for 15 min and the separated plasma then fractionated and stored at −20°C until hormone assay.

Hormones in the plasma samples including testosterone (T), free testosterone (FT), dihydrotestosterone (DHT), estradiol (E2), and sex hormone binding globulin (SHBG) were analyzed by ELISA using Diagnostics Biochem Canada Inc. (Ontario, Canada), and the instrument ELISA Microplate Reader, model: Sunrise (Tecan Austria GmbH Group, Austria, Gordig). The intra-assay coefficients of variation (CVs) % and assay sensitivity were 6.8 and 0.022 ng/ml for T; 7.8 and 0.17 pg/ml for FT; 7.4 and 6 pg/ml for DHT; 7.6 and 10 pg/ml for E2; 5.8 and 0.1 nmol/L for SHBG, respectively.

### Statistical analysis

Data are expressed as mean ±SD and a Statistical Package for the Social Sciences [(SPSS 18.0), New York: McGraw-Hill] was used to perform all comparisons and independent sample T-test was used to compare quantitative variables between the study groups.

A P-value of less than 0.05 was considered significant for the differences.

## Results

The mean ± SD of age in the control subjects and patients were 39.0±8.0 and 45.0±10.0 years, respectively (P<0.004).

The comparison of serum hormonal levels of the subjects in both groups is shown in table 1.

**Table 1:** Comparison of gonadal sex hormone concentrations of the controls and patients.

Group▸	Control (n = 46)	Patients (n = 40)	P value
Variable▾			
Testosterone (ng/ml)	2.41±1.06	3.30±2.50	0.03
Free Testosterone (Pg/ml)	3.43±1.10	4.59±3.82	0.05
Dihydrotestosterone (Pg/ml)	200.60±86.10	270.0±206.80	0.04
Estradiol (Pg/ml)	28.03±22.50	53.40±22.60	0.00
Sex hormone binding globulin (nmol/l)	28.64±10.36	46.6±18.50	0.00

Values are expressed as mean ± SD.

A significant difference was observed between patients and the control subjects regarding serum testosterone, free testosterone, dihydrotestosterone, estradiol, and sex hormone binding globulin.

Based on the results of sex hormone investigation, a higher androgen level was diagnosed in patients.

These results indicate that there is a possibility of testosterone, free testosterone, and dihydrotestosterone involvement in the pathogenesis of renal stones and show that high level of androgens may have a substantial pathogenic role and may partly enhance the kidney stone formation.

## Discussion

Although the pathophysiologic mechanisms of some disorders, such as hyperparathyroidism, have been elucidated, the actions of other hormones on urolithiasis remain elusive.

In the present study, a significant higher level of serum testosterone, free testosterone, dihydrotestosterone in patients indicates a potential role for the gonadal steroids in the pathogenesis of urolithiasis and a positive relationship for incidence of kidney stones in males. The association between serum testosterone and urolithiasis has yet received only limited attention. The cause of this predisposition in males is currently unknown, and the reason is obscure. Therefore, data presentation and further investigation on the relation between male steroids and urolithiasis is of importance and should be considered in evaluation of the etiology of the disease. In some animal studies, it has been shown that the administration of testosterone increases urinary oxalate excretion and enhances the formation of calcium oxalate stones [9] and most of animal studies used castrated or ovariectomized animal models [5]. Menopausal women might have an increased potential in higher urinary calcium and calcium oxalate saturation as compared to premenopausal women [8].

Testosterone is known to increase the hepatic levels of glycolic acid oxidase (GAO) [12], and thus may lead to an increase in hepatic synthesis of this enzyme, resulting in hyperoxaluria, which in turn may be responsible for the increased predisposition to calcium oxalate urolithiasis [11]. It promotes stone formation by suppressing renal osteopontin expression and increasing urinary oxalate excretion [9]. Active dihydrotestosterone (DHT) is produced from testosterone by cytosolic enzyme, 5α-reductase and has been believed to be partially responsible for exaggerated hyperoxaluria observed in the rat ethylene glycol model of urolithiasis [13].

It is known that the major circulating androgen in males is testosterone, and about 98% of testosterone molecules are bound to proteins in the blood, principally to sex hormone-binding globulin (SHBG) and also to albumin and cortisol-binding globulin, as well. It is assumed that bound hormones cannot exit blood capillaries and are therefore not bioavailable [15].

The free hormone hypothesis states that the biological activity of a given hormone is affected by its unbound (free) rather than protein-bound concentration in the plasma. This free testosterone is considered the biologically active form of the hormone, as this portion of the hormone can interact at the target tissue receptors [16]. In cross-sectional and longitudinal studies of men aged 30 or 40 years and above, total, bioavailable and free testosterone concentrations fall with increasing age with a steeper decline in bioavailable and free compared with total testosterone concentrations [17]. In older men above the age of 65 or 70 years, the changes in total testosterone are overshadowed by more significant declines in free testosterone levels [18]. Given the higher plasma level of free testosterone and dihydrotestosterone and their diverse roles seen in association with kidney stone incidence, the hypothesis of hyperandrogens is likely to be valid for stone formation. Jyoti Nath et al. (2013) reported a higher serum free and total testosterone, and 24 hours of urinary oxalate in male stone formers with a positive correlation between serum testosterone with urinary oxalate [19]. Moreover, in another study, although no significant difference was found for testosterone between the male active renal calcium stone formers and control groups, serum testosterone was related to higher urinary excretion of uric acid in patients and to higher urinary excretion of oxalate in the control group, representing the possibility of testosterone involvement in the pathogenesis of renal stones [4]. In a case report, the association between serum gonadal steroids and urolithiasis in a 38-year old patient was confirmed after twice repeated estimation of testosterone, free testosterone, dihydrotestosterone, estradiol, and sex hormone binding globulin revealed hyperandrogenicity [20].

Polycystic ovary syndrome (PCOS), one of the most frequent endocrine disorders of women in the reproductive age which is characterized with clinical or biochemical evidence of hyperandrogenism may trigger the urinary stone formation and is known to be a risk factor in the formation of urinary stone disease [21]. The post menopausal female condition of low estrogen resembles the male hormonal status and the protective role of estradiol in premenopausal women compared with menopausal women who might have an increased potential for urinary stone formation is speculated [8].

Recently, it was reported that naturally postmenopausal women who have higher remaining estradiol levels appear less likely to suffer from kidney calcium oxalate stones [22].

Although the observed significant increase in plasma estradiol in patients indicates a higher rate of conversion of total testosterone to estradiol in the testosterone metabolic pathway, it seems that even such a significant increased level is not strong enough to prevent stone formation in males. To our knowledge, this data is complementary to previous reported studies [6], [19] to estimate the concentrations of active androgens with estradiol and SHBG concentrations, and to characterize the association of high androgens in the pathogenesis of urolithiasis in adult urolithic men. The two aforementioned studies found a positive correlation for free and total testosterone in relation to calcium oxalate stones, too.

In conclusion, a positive relationship exists between high plasma androgen concentrations and incidence of kidney stones, which attributes a potential role for the gonadal steroids in the pathogenic mechanism in male idiopathic urolithiasis.
